# Translating Observational Data Into Public Health Action: Reducing Early Childhood Caries Burden Related to Socioeconomic Disparities

**DOI:** 10.1111/cdoe.70064

**Published:** 2026-03-23

**Authors:** An T. M. Dao, Loc G. Do, Helena S. Schuch, Huy V. Nguyen, Diep H. Ha

**Affiliations:** ^1^ School of Dentistry, Faculty of Health and Behavioural Sciences The University of Queensland St Lucia Queensland Australia; ^2^ Cuu Long University Vinh Long Vietnam; ^3^ Division of Epidemiology, Department of Population and Quantitative Health Sciences UMass Chan Medical School Worcester Massachusetts USA; ^4^ Health Innovation and Transformation Centre Federation University Australia Ballarat Victoria Australia

## Abstract

**Objectives:**

The “valley of death” between research and practice remains a major challenge. The five‐stage Translational Research framework (TR1‐TR5) provides a pathway, moving evidence into real‐world impact. Traditionally, Randomised Controlled Trials (RCTs) dominate TR stages, yet they are often impractical in public health. Longitudinal observational studies (LOSs), combined with advanced causal statistics, can emulate RCTs. Using the relationship between socioeconomic status (SES) and early childhood caries (ECC) as a case study, where SES is a complex, upstream determinant lacking trial evidence, this study illustrates how LOSs paired with causal statistics, within the TR framework, can generate policy‐relevant action, highlighting a promising yet underused strategy.

**Methods:**

This paper first reviewed causal inference gaps identified in a published scoping review of 85 LOSs (TR1), focusing on a subset of 20 studies presented in this paper that examined the relationship between SES and dental caries. The review analysed SES construction, gaps in SES‐oral health behaviour‐caries pathways, and the use of advanced causal statistics. Findings from TR2 (published) were then summarised to highlight key modifiable mediators linking SES and ECC, followed by TR3‐4 (published), which examined interventions targeting these mediators across populations. Finally, this paper presented TR5 for the first time, extending the analysis to estimate population‐level impact metrics.

**Results:**

TR1 highlighted that most LOSs remained focused on associations rather than causal inference. SES construction was inconsistent and the mechanisms linking SES to dental caries have not been comprehensively examined. TR2 identified free sugar intake (FSI) as a key modifiable mediator. TR3‐4 emphasised on reducing FSI to < 10% lowered ECC risk in the general population and suggested < 5% for the high‐risk groups. TR5 highlighted that stricter FSI thresholds produced substantial population‐level gains.

**Conclusion:**

The TR framework supported by advanced causal statistics enables translating LOSs into effective public health evidence in the absence of RCTs.

## 1 Introduction

Despite major advances in research, a persistent gap, known as the “valley of death” in evidence‐based healthcare, remains between generating evidence and achieving real‐world health impact [1]. Translational research (TR) is an approach to research that focuses on turning scientific discoveries into practical applications [2], offers a structured process to bridge this gap [3].

In clinical medicine, TR aims to integrate proven interventions into routine practice [4]. Its goal is not only to advance innovations into clinical trials but also to ensure their adoption for the benefit of society [5]. For instance, if a new drug is shown to be safe and effective, translational medicine emphasises changing prescribing patterns and supporting patient uptake [6].

In public health, TR has a broader scope, targeting population‐level outcomes such as improved wellbeing or reductions in morbidity and mortality [6]. To this end, the TR framework for public health outlines a process of five stages: from basic science and identification of modifiable determinants, through intervention evaluation across populations, to evidence synthesis that informs health strategies [6].

Traditionally, TR has relied on randomised controlled trials (RCTs). However, RCTs are not always feasible or ethical in public health, particularly when timely or population‐wide evidence is needed [7]. By contrast, longitudinal observational studies (LOSs), when supported by advanced causal inference methods [8], such as causal mediation analysis (CMA) [9], G‐computation, and Targeted Maximum Likelihood Estimator (TMLE) [10], can emulate RCTs and generate robust, policy‐relevant evidence [1, 8]. These methods apply the counterfactual framework: CMA estimates mediating effects by holding exposure and mediator at defined thresholds [11]. G‐computation predicts outcomes under different counterfactual exposures and compares their population averages, while TMLE integrates outcome and exposure models to improve efficiency and reduce bias [10]. These approaches are particularly valuable for addressing causation public health questions where trials are impractical but causal estimates are urgently needed. For instance, the relationship between socioeconomic status (SES) and early childhood caries (ECC), a condition affecting nearly half of children worldwide [12, 13], illustrates this challenge.

While SES is recognised as a major upstream determinant of ECC [14], it is difficult to intervene on directly because it is multidimensional, not easily modified, and lacks trial‐based evidence. Applying the counterfactual approach to LOS data within the TR framework therefore offers a promising but underused strategy to reduce SES‐related inequalities in ECC, especially in the absence of RCTs [15].

Using SMILE birth cohort data, with SES measured at baseline and ECC at later stages, this paper drew on longitudinal observational study (LOS) data supported by advanced causal inference methods. The paper aimed to discuss the application of the translational research framework in public health to bridge the gap between scientific evidence and real‐world practice in addressing a public health problem.

## 2 Methods

### 2.1 Study Designs

**FIGURE 1:**
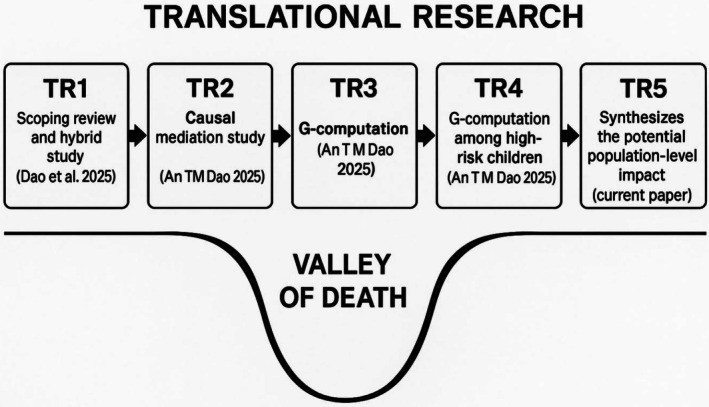
The five stages of translational research.

This paper synthesised five interlinked studies conducted within a TR framework (Figure 1) to advance causal inference in dental caries research. Rather than reporting new data collection, the manuscript integrated evidence across publications (TR1 to TR4), and current analyses (TR1 subset and TR5) to demonstrate the stepwise progression from identifying knowledge gaps to estimating population‐level impact.

TR1 was a scoping review of 85 LOSs, published elsewhere [15], which mapped gaps in causal inference. A TR1 subset of 20 LOSs (Appendix Table S1) examining socioeconomic status (SES) and dental caries was re‐analysed here to provide domain‐specific insights of causal mechanism and causal statistics that guided subsequent stages.

Building on TR1, a hybrid study published elsewhere [16] developed a multidimensional robust SES variable. This predictor was used to model ECC, defined as the presence of one or more carious lesions, white spot lesions, tooth loss due to caries, or filled surfaces due to caries in the primary teeth of a child under 6 years old [17]. The SES variable served as the exposure/predictor in TR2 and as a confounder in TR3‐TR5. TR2, a causal mediation analysis published elsewhere, estimating natural indirect effects (NIEs), identified the most impactable mediator linking SES and ECC at age five [18].

TR3‐4 applied G‐computation to estimate the effects of reducing the level of the identified modifiable mediator on ECC in both general population and the high‐risk group (children who exceeded FSI intake of 10% or 5% of EER at age two and developed dental caries by age five), accounting for confounders. Negative binomial regression was fitted in which FSI at age two was the exposure used to predict ECC at age five, accounting for baseline and time‐varying confounders (Appendix Figure S1). These analyses were published elsewhere [19] Their designs are summarised here to maintain continuity in the translational pathway.

Specifically, the analyses estimated how dmfs scores at age five would have changed if the entire preschool population had reduced their FSI intake from ≥ 10% or ≥ 5% of total energy to below these thresholds. Causal effect measures included the absolute reduction (AR), the difference in mean dmfs scores between populations consuming sugar above versus within recommended thresholds, and the attributable fraction among the exposed (AFE), representing the proportion of dental caries among children with FSI ≥ 10% or ≥ 5% of energy intake attributable to the exposure (Appendix Figure S2). By conditioning the entire population to a specified level of exposure versus non‐exposure, G‐computation provides intention‐to‐treat (ITT)‐like causal contrasts, rather than restricting analysis to participants with complete data as in per‐protocol approaches [20].

**FIGURE 2:**
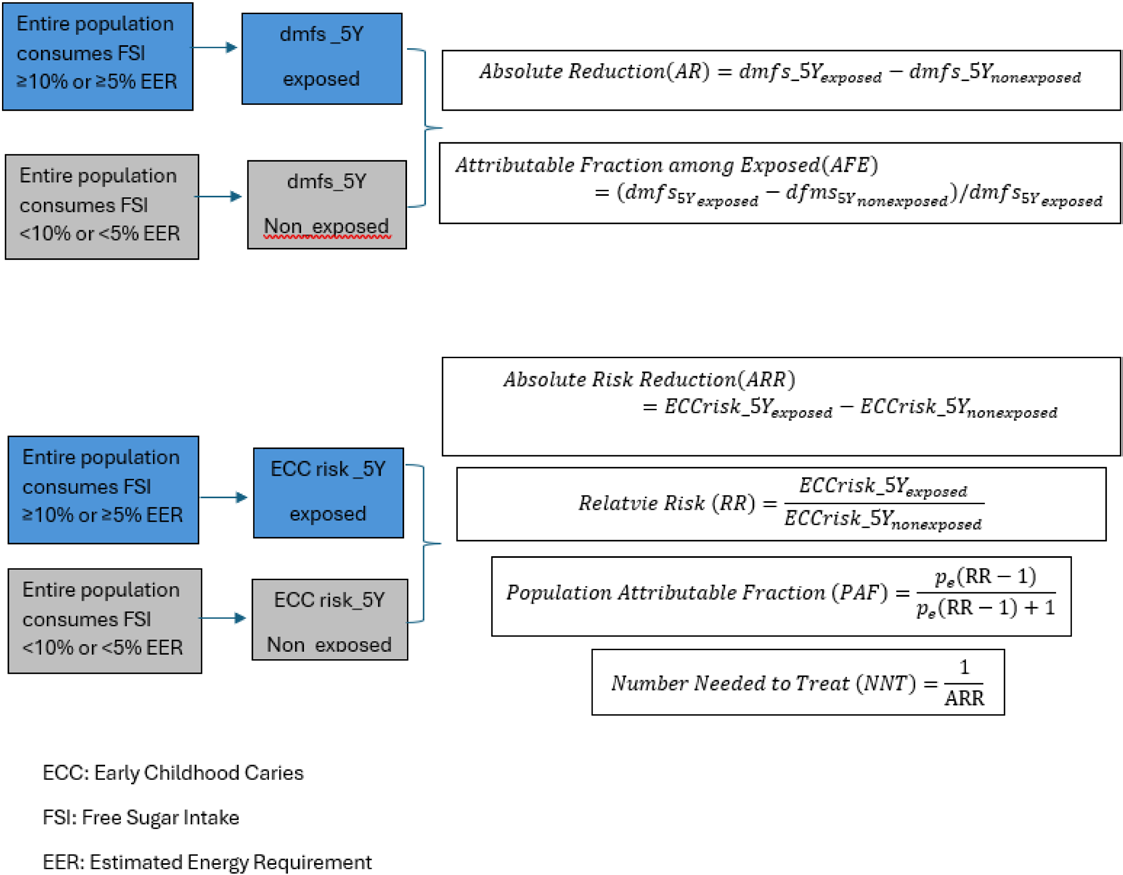
Metrics estimated from translational research Stage 3 to Stage 5.

TR5 analyses extended previous analyses by estimating population‐level impact metrics, including the absolute risk reduction (ARR, the difference in risk between groups), the population attributable fraction (PAF, the proportion of cases in the population attributable to the exposure), and the number needed to treat (NNT, the number of children who would need the intervention to prevent one additional case of dental caries) (Figure 2).

The current work primarily focuses on two key components of this TR pathway: (i) the domain‐specific synthesis of 20 LOSs within the TR1 subset and (ii) the TR5 population‐level analysis. These findings were situated within the broader translational research pathway.

### 2.2 Data Source

The data for TR1 were drawn from 85 LOSs identified in five major databases, focusing on determinants of dental caries published from 2012 to 2024 [15]. For the hybrid study and TR2 to TR5, the data were obtained from the *Study of Mothers' and Infants' Life Events Affecting Oral Health* (SMILE) [21], funded by NHMRC Project Grant #1046219 (2013–2016) and approved by relevant ethics committees [22].

SMILE recruited 2182 mother–infant dyads from Adelaide's three largest public hospitals between mid‐2013 and mid‐2014. Mothers were enrolled within 48 h postpartum with informed consent and received participation incentives. Full recruitment details are published elsewhere [21]. Children and their caregivers were followed up at 3, 6, 12, 24, and 60 months to collect longitudinal data on oral health and related exposures.

For TR2‐TR5, free sugar intake (FSI) measured at age two was used to predict dmfs scores at age five. At age two, parents completed a 99‐item Food Frequency Questionnaire (FFQ), which had been validated against 24‐h recalls [22]. FSI was extracted from relevant items, converted to mg/day, and outliers above the 99th percentile were excluded. For G‐computation, FSI was categorised as binary using thresholds of 10% and 5% of estimated energy requirements (EER) based on Nutrient Reference Values for Australia and New Zealand [23]. Using daily EERs of 4200 kJ for girls and 4400 kJ for boys (17 kJ ≈1 g sugar), thresholds corresponded to: 24.7 g/day (girls) and 25.9 g/day (boys) for 10%, and 12.4 g/day (girls) and 12.9 g/day (boys) for 5%.

The dmfs scores were obtained through a standardised oral epidemiology examination conducted by trained examiners when children reached ages 2 and 5 [22]. Each tooth surface affected by decay, missing due to caries, or filled was scored, and the cumulative dmfs score was used as a continuous outcome.

Twenty‐one SES‐related variables were measured at baseline, corresponding to 12 key indicators used to construct an SES composite; details of its construction are reported elsewhere [16]. Other confounders included plaque, assessed at ages two and five and categorised into four levels (0 = no plaque, 1 = thin plaque detected with probing, 2 = visible plaque without probing, 3 = abundant plaque); for analyses, plaque was dichotomised as “invisible” (0–1) versus “visible” (2–3) [22]. Dental visit patterns were captured at age five from parent reports of all dental visits during the first 5 years, with the total number of visits summed for analysis [22].

### 2.3 Statistics

For the review of the 20 LOSs examining SES–dental caries relationships, the percentage of studies that fully addressed time‐varying variables, defined confounders, visualised directed acyclic graphs (DAGs), and applied counterfactual approaches for causal inference was estimated.

In the hybrid SES study, decision tree analysis (DTA) and principal component analysis (PCA) were combined to create SES composites. Zero‐inflated regression models and root mean squared error (RMSE) were used to evaluate predictive accuracy, with the most robust composite identified by comparing model performance across SES versions [24].

The TR2 applied causal mediation analysis (CMA), incorporating mediator, outcome and counterfactual models, accounting for potential confounders, to estimate the natural indirect effects (NIE) and the proportion mediated [18]. A factor was identified as mediator if all three models were statistically significant [11].

The TR3‐TR5 used G‐computation, initiating with a negative binomial regression with FSI age two as predictor and ECC age five as outcome, first unadjusted, then sequentially adjusted for baseline SES [15, 25], child's sex [26, 27], and time‐varying variables, ECC at age two [28], dental visits pattern during the first 5 years [29], and plaque index at ages two and five [30]. Based on the model parameters, counterfactual outcomes were estimated under two scenarios: if the entire population consumes FSI at ≥ 10%/≥ 5% versus < 10%/< 5% of EER. From these two counterfactuals, AR, ARR, AFE, PAF, and NNT were calculated for different scenarios.

## 3 Results

The review of 85 longitudinal observational studies (LOSs) on determinants of dental caries for TR1 showed that, although these studies provided valuable insights, most focused‐on associations rather than causal inference, with none fulfilling all four essential steps of causal analysis. None formulated a causal question using a counterfactual framework to estimate the average potential outcome, i.e., to answer the question *“What would the outcome have been if the entire population had reduced exposure to a specific risk factor?”* or examined mechanisms linking specific exposures to outcomes through causal pathway modelling. Only a minority applied Directed Acyclic Graphs (DAGs) to visualise relationships among variables and account for confounders. While most LOSs collected time‐varying data, few fully integrated these data into model development. Over half struggled to implement statistical approaches that were both robust and consistent with their DAG specifications, limiting their ability to appropriately manage confounding from evolving data relationships [15].

**FIGURE 3:**
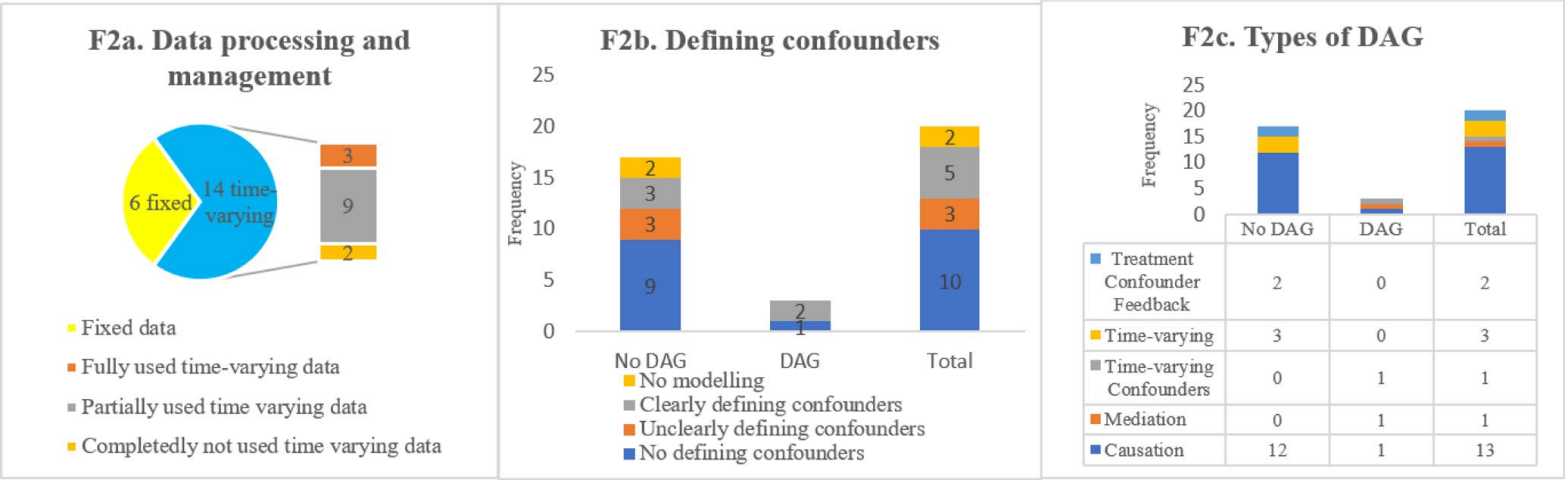
The causal approach assessing the association between socioeconomic status and dental caries.

**FIGURE 4:**
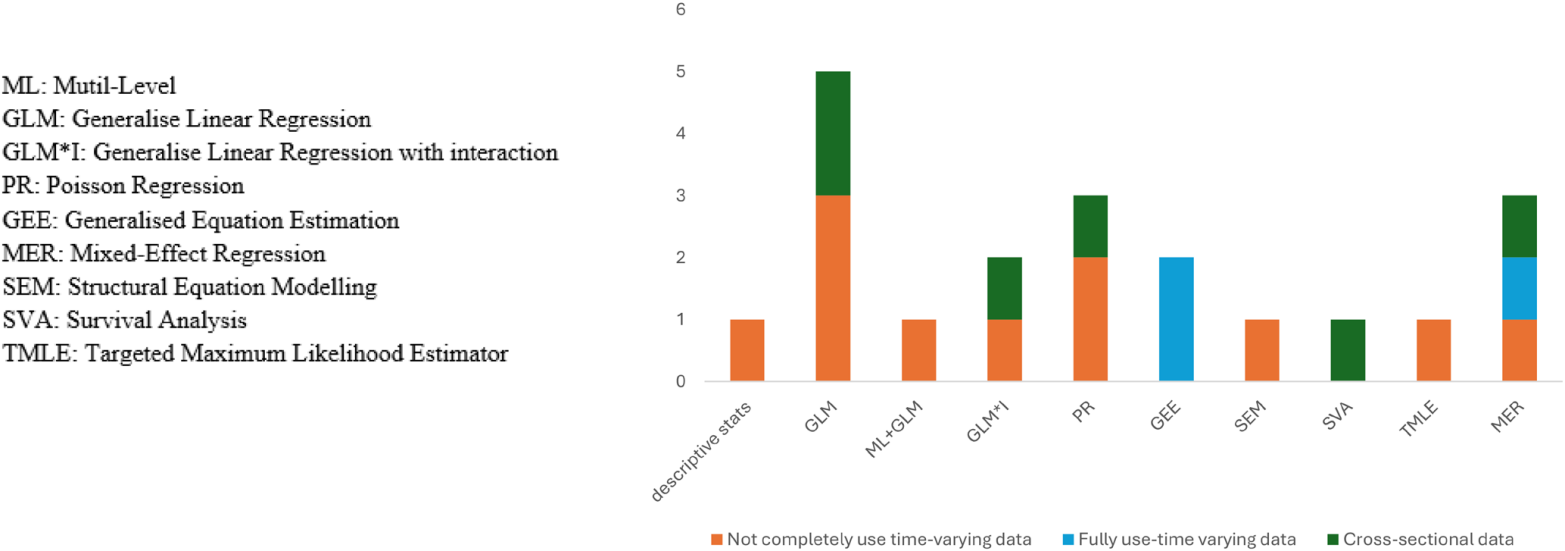
Alignments between directed acyclic graphs and causal statistics.

The subset re‐analysis of 20 longitudinal observational studies (LOSs) examining the SES‐dental caries relationship echoed these findings (Figures 3 and 4; Appendix Table S2). It further showed that SES indicators were inconsistently constructed as predictors in modelling and were often not specifically tailored to dental caries outcomes (Appendix Figure S3). Notably, among the three studies that visualised Directed Acyclic Graphs (DAGs), none comprehensively examined the SES‐oral health behaviours‐dental caries pathway. Costa et al. employed structural equation modelling (SEM) incorporating oral health behaviours as mediators to examine two pathways‐SES‐dental caries and social support–dental caries [31]. However, a more comprehensive causal pathway analysis remains needed, as this study constructed two latent variables (SES and social support) that may represent overlapping dimensions of socioeconomic status, defined as one's access to social and economic resources [32]. Moreover, the SEM methods used were not designed for causal inference. Similarly, Matsuyama et al. applied targeted maximum likelihood estimation (TMLE), which appropriately addressed time‐varying confounders but did not include oral health behaviours as mediators [33]. Vieira‐Andrade et al. used a hierarchically adjusted Poisson regression model guided by a causal DAG [34]; however, this single‐level regression approach does not constitute a fully causal analytic framework.

### 3.1 Results From the TR2‐TR5 Analyses

Among the 2182 recruited children, data completeness varied: 1047 for FSI (excluding 10 extreme values), 1878 for SES, 824 for dental visits, 1394 for gender, and 879–1041 for clinical measures (dmfs scores and plaque indices) at ages two and five. FSI at age two was right‐skewed (median 22.6 g/day), with 44.3% and 75.2% exceeding the 10% and 5% EER thresholds, respectively, and no differences by sex. ECC at age five was also right‐skewed (median 0), with 32.5% of children affected. Baseline SES showed an approximately normal distribution. Using these data, TR2 indicated that FSI at age two identified as the key mediator between SES and ECC.

Building on these foundations, TR3‐4 employed a subset of 541 children of the SMILE study who had complete data for all study variables, child's gender, SES composite, FSI at age two, ECC at age two and five, dental visits pattern during the first 5 years, and plaque index at ages two and five to conduct G‐computation. TR3‐4 demonstrated the preventive impact of reducing free sugar intake (FSI) on early childhood caries (ECC) at age five. Reducing FSI to below 10% of total energy requirement (EER) was associated with more than an 80% reduction in decayed, missing, and filled surfaces (dmfs) in the general population and a 75% reduction among high‐risk children. Further reduction below 5% yielded up to a 97% to 99% risk reduction, respectively (Appendix Figure S4).

In this analysis, high‐risk children were defined as those with FSI > 10% (*n* = 78) or > 5% (*n* = 133) of EER who developed ECC by age five. In this subgroup, FSI and ECC levels were substantially higher than the overall sample (median FSI: 41.2 g/day for ≥ 10% EER, 30 g/day for ≥ 5% EER), whereas distributions in the 541‐child subgroup were otherwise comparable to the full cohort.

TR5 extended these findings to assess the broader population‐level impact in different scenarios and consistently showed that a greater population‐wide reduction in FSI leads to a greater decrease in caries risk. Specifically, if FSI were reduced to below 10% of total energy intake, the estimated PAF was 10% and the NNT was 15. In contrast, further reduction below 5% yielded a PAF of 21% and an NNT of 9. Noticeably, when FSI was reduced to either < 10% or < 5%, both in the general and high‐risk groups, the absolute risk reduction (ARR) and population attributable fraction (PAF) nearly doubled, while the number needed to treat (NNT) was halved, compared with scenarios in which only half the population achieved these FSI reductions (Table 1).

**TABLE 1:** Population‐level impact metrics of intervention on reducing Free Sugar Intake.

	General population					High‐risk population				
	Risk of dmfs > 0	ARR	RR	PAF	NNT	Risk of dmfs > 0	ARR	RR	PAF	NNT
FSI at 10% EER										
Half pop consuming FSI < 10%	0.31	0.03	1.23	0.05	29.95	0.33	0.03	1.09	0.07	29.41
Whole pop consuming FSI < 10%	0.28	0.07	1.25	0.10	15.01	0.29	0.07	1.24	0.15	14.71
Whole pop consuming FSI ≥ 10%	0.35					0.36				
FSI at 5% EER										
Half pop consuming FSI < 5%	0.28	0.06	1.21	0.09	15.50	0.28	0.07	1.25	0.15	15.45
Whole pop consuming FSI < 5%	0.22	0.13	1.55	0.21	8.33	0.22	0.13	1.59	0.31	7.73
Whole pop consuming FSI ≥ 5%	0.34					0.35				

Abbreviations: ARR, Absolute Risk Reduction; EER, Estimated Energy Requirement; FSI, Free Sugar Intake; NNT, number of people who need to be treated to prevent one additional adverse event; PAF, Population attributable fraction; Pop, population; RR, Relative Risk.

## 4 Discussion

This paper illustrates how applying the five‐stage translational research (TR) framework in public health can transform longitudinal observational study (LOS) data into robust, policy‐relevant evidence, even in the absence of randomised controlled trials (RCTs). When implemented thoroughly from TR1 to TR5, this framework bridges the gap between established scientific evidence and real‐world action to address complex public health problems. Using the relationship between socioeconomic status (SES) and dental caries as a case study, the study highlights that progressing through all five TR stages is essential for comprehensively addressing public health challenges, with each stage representing a successive step in translating LOS data into actionable insights.

TR1 typically begins with a well‐recognised scientific issue that remains abstract in terms of practical solutions. In this case, the focus was on the long‐established influence of socioeconomic status (SES) on early childhood caries (ECC). The review revealed critical gaps that LOSs have largely remained focused on associations rather than causal inference, as none fulfilled all four essential steps of causal analysis. The use of SES indicators as predictors has been inconsistent, often derived either from knowledge‐based or data‐driven approaches that were not specifically tailored to dental caries outcomes.

Moreover, the mechanisms linking SES to dental caries have not been comprehensively examined in terms of both causal pathway frameworks and causal pathway statistics. To inform actionable public health strategies aimed at reducing SES‐related inequalities in dental caries, robust causal evidence is needed. Future longitudinal studies should therefore:

1. construct SES predictors that integrate both data‐driven and theory‐informed indicators;
2. incorporate modifiable oral health behaviours as mediators within mediation DAGs; and
3. apply causal pathway statistics consistent with hypothesised causal DAGs.

Building on these findings, TR1 later stage developed a robust SES composite predictor to examine mechanisms linking SES and ECC in TR2. This stage identified free sugar intake (FSI) at age two as a key modifiable mediator, based on the statistical significance of its natural indirect effect (NIE). Consequently, TR3‐4 assessed the potential impact of reducing free sugar intake (FSI) below WHO‐recommended thresholds on the burden of early childhood caries (ECC) using metrics such as Absolute Reduction (AR) and the Attributable Fraction among the Exposed (AFE) across the general preschool population and a high‐risk subgroup. By comparing the effects achieved at the two FSI reduction thresholds (10% vs. 5%), estimated reductions ranged from 84% to 97% in the general population and 75%–99% in the high‐risk group. These findings support that a 10% reduction may be appropriate for the general population, whereas a 5% reduction would yield greater benefit among high‐risk children. TR5 took this analysis a step further by providing actionable insights for decision‐makers. The PAF estimations inform that if all preschool children reduced their free sugar intake to below 10% or 5% of total energy intake, approximately one or two in 10 cases of early childhood caries (ECC) could be averted, respectively. Among high‐risk children, the potential benefit was even greater—two to three in 10 cases could be prevented. Similarly, the NNT estimates indicate that reducing FSI to below 10% or 5% of total energy intake in 16 or 9 children, respectively, would prevent one additional case of ECC in the general preschool population. For the high‐risk group, the corresponding figures were 15 and 8, respectively (Table 1).

TR5 estimated a relative risk of 1.25 for FSI exceeding 10%, which corresponds to a 7% absolute risk reduction (ARR) and a 10% population attributable fraction (PAF). This estimated PAF is consistent with findings from other studies on behavioural risk factors. For instance, non‐regular exercise has been linked to 13% of stage‐1 hypertension cases, insufficient physical activity to 25% of stage‐2 hypertension, and low energy expenditure to 8.5% of cardiovascular events [35]. Additionally, type 2 diabetes has been attributed to 3%–7% of cases in Finland and 13%–30% in the US [36], while high sodium intake accounts for approximately 20% of cardiovascular deaths in Shandong, China [37]. Scaled to 286 998 Australian births in 2023 [38], reducing FSI below 10% could prevent ~28 699 ECC cases (~19 000 by NNT, 1 case per 15 children), with even greater benefits at the 5% threshold. These comparisons indicate that our PAF lies within the expected range of preventable burden from behavioural risk factors, supporting the public health relevance of reducing FSI.

Collectively, these five stages demonstrate the power of the TR framework to move from theoretical evidence to actionable public health strategies. By leveraging LOS data and advanced causal inference methods, robust evidence can be generated to guide policy and intervention decisions, even without the presence of RCTs. However, several methodological considerations are essential to ensure the robustness of such evidence.

First, attrition bias is a common challenge in LOSs. The SMILE cohort, for example, experienced attrition by year five, with participants having higher SES than at baseline. Strategies to address this issue should be considered early in study design, such as applying weighting methods for participants likely to be lost to follow‐up or selecting additional sampling sites to compensate for attrition. The SMILE study mitigated this risk by recruiting exclusively from public hospitals serving socioeconomically disadvantaged populations [39]; and oversampling lower‐SES mothers [22].

Second, while LOSs can emulate RCTs using counterfactual approaches such as G‐computation, these methods cannot fully eliminate potential biases from unmeasured confounding, especially in complex exposures such as SES. The validity of G‐computation estimates depends on the assumption of no unmeasured confounding and correct model specification [10]. To mitigate these limitations, we used directed acyclic graphs (DAGs) to identify and adjust for relevant covariates, incorporated time‐varying determinants, and validated results through sensitivity analyses including k‐fold cross‐validation, E‐value estimation, and dose–response assessment. However, unlike RCTs, residual confounding related to unmeasured socioeconomic or contextual factors cannot be fully ruled out.

Finally, when applying counterfactual approaches, researchers must balance methodological rigor with interpretability to generate evidence that is both credible and actionable for public health practice. In this study, G‐computation was chosen over targeted maximum likelihood estimation (TMLE) as it provides a more straightforward and parsimonious modelling framework. Furthermore, within the G‐computation analyses, interaction terms were not included to maintain simplicity and ensure that the findings could be easily interpreted and translated into actionable insights for decision‐makers [10]. Once the framework and findings are well understood, future studies could extend this work using TMLE to explore whether the effects of sugar intake on caries differ across socioeconomic groups or other modifying factors.

While FSI has been identified as a key mediator of the SES‐ECC relationship in South Australian children, future strategies should test sugar reduction across diverse sociocultural contexts, including low‐ and middle‐income countries, to determine “what works, for whom, and in what context” [40]. Beyond TR5, effective interventions must also address intersectoral and structural pathways. As shown in salt reduction, RCTs demonstrated efficacy [41], but the real‐world impact in Western countries was limited by processed food environments [42]. Reducing ECC disparities requires moving beyond individual behaviour change toward population‐wide, structural approaches [40].

In conclusion, the TR framework, combined with advanced causal inference methods, can translate longitudinal observational data into rigorous, policy‐relevant evidence, even in the absence of RCTs. This approach supports equitable public health interventions, as illustrated by our study on child oral health and historically by interventions for sudden infant death syndrome (SIDS), where impactful strategies arose from observational insights rather than clinical trials [43].

## Funding

This work was supported by National Health and Medical Research Council (Grant 1046219).

## Conflicts of Interest

The authors declare no conflicts of interest.

## Supporting information


**Table S1:** Twenty longitudinal observational studies examining association between SES and dental caries (2012–2024).
**TABLE S2:** Alignment types of the Directed Acyclic Graphs (DAGs) and statistical methods.
**FIGURE S1:** Directed Acyclic Graphic, visualising relationship between Free Sugar Intake age two and Early Childhood Caries at five.
**FIGURE S2:** G‐computation framework, estimating Absolute Reduction and Attributable Fraction among Exposure.
**FIGURE S3:** SES relevant items used in longitudinal observational studies predicting dental caries from 2012 to 2024.
**FIGURE S4:** Missing, filled surfaces (dmfs) scores reduction at age five in the conditions of Free Sugar Intake age two below 10% and 5% of total estimated energy requirements.

## Data Availability

The data that support the findings of this study are available on request from the corresponding author. The data are not publicly available due to privacy or ethical restrictions.

## References

[1] “Traversing the Valley of Death,” Nature Reviews Bioengineering 1, no. 12 (2023): 875.

[2] K. M. Narayan , E. W. Gregg , M. M. Engelgau , et al., “Translation Research for Chronic Disease: The Case of Diabetes,” Diabetes Care 23, no. 12 (2000): 1794–1798. 11128355 10.2337/diacare.23.12.1794

[3] A. Nazarian , “Translational Science Research: Bridging the Gap Between Lab and Life for a Better Tomorrow,” Journal of Medicinal and Organic Chemistry 6, no. 5 (2023): 119–120.

[4] E. M. Ginexi and T. F. Hilton , “What's Next for Translation Research?,” Evaluation & the Health Professions 29, no. 3 (2006): 334–347. 16868341 10.1177/0163278706290409

[5] V. Mahalmani , S. Sinha , A. Prakash , and B. Medhi , “Translational Research: Bridging the Gap Between Preclinical and Clinical Research,” Indian Journal of Pharmacology 54, no. 6 (2022): 393–396. 36722550 10.4103/ijp.ijp_860_22 PMC10043823

[6] D. Ogilvie , P. Craig , S. Griffin , S. Macintyre , and N. J. Wareham , “A Translational Framework for Public Health Research,” BMC Public Health 9, no. 1 (2009): 116. 19400941 10.1186/1471-2458-9-116 PMC2681470

[7] L. J. Finney Rutten , J. L. Ridgeway , and J. M. Griffin , “Advancing Translation of Clinical Research Into Practice and Population Health Impact Through Implementation Science,” Mayo Clinic Proceedings 99, no. 4 (2024): 665–676. 38569814 10.1016/j.mayocp.2023.02.005

[8] M. A. Hernan , I. J. Dahabreh , B. A. Dickerman , and S. A. Swanson , “The Target Trial Framework for Causal Inference From Observational Data: Why and When Is It Helpful?,” Annals of Internal Medicine 178, no. 3 (2025): 402–407. 39961105 10.7326/ANNALS-24-01871 PMC11936718

[9] K. Imai , L. Keele , and D. Tingley , “A General Approach to Causal Mediation Analysis,” Psychological Methods 15, no. 4 (2010): 309–334. 20954780 10.1037/a0020761

[10] A. Chatton , F. le Borgne , C. Leyrat , et al., “G‐Computation, Propensity Score‐Based Methods, and Targeted Maximum Likelihood Estimator for Causal Inference With Different Covariates Sets: A Comparative Simulation Study,” Scientific Reports 10, no. 1 (2020): 9219. 32514028 10.1038/s41598-020-65917-x PMC7280276

[11] J. J. M. Rijnhart , S. J. Lamp , M. J. Valente , D. P. MacKinnon , J. W. R. Twisk , and M. W. Heymans , “Mediation Analysis Methods Used in Observational Research: A Scoping Review and Recommendations,” BMC Medical Research Methodology 21, no. 1 (2021): 226. 34689754 10.1186/s12874-021-01426-3 PMC8543973

[12] S. E. Uribe , N. Innes , and I. Maldupa , “The Global Prevalence of Early Childhood Caries: A Systematic Review With Meta‐Analysis Using the WHO Diagnostic Criteria,” International Journal of Paediatric Dentistry 31, no. 6 (2021): 817–830. 33735529 10.1111/ipd.12783

[13] L. G. Do and A. J. Spencer , Oral Health of Australian Children: The National Child Oral Health Study 2012–14 (University of Adelaide Press, 2016).

[14] S. A. Fisher‐Owens , S. A. Gansky , L. J. Platt , et al., “Influences on Children's Oral Health: A Conceptual Model,” Pediatrics 120, no. 3 (2007): e510–e520. 17766495 10.1542/peds.2006-3084

[15] A. T. M. Dao , L. G. do , N. Stormon , M. Dhanapriyanka , and D. H. Ha , “Causal Analyses in Longitudinal Observational Studies in Oral Health: A Scoping Review,” Community Dentistry and Oral Epidemiology 53, no. 1 (2024): 7–16. 39248439 10.1111/cdoe.13002 PMC11754146

[16] A. T. M. Dao , L. G. do , N. Stormon , H. V. Nguyen , and D. H. Ha , “Enhancing Socioeconomic Status Prediction for Cavities: A Hybrid Method,” Journal of Dental Research 104 (2025): 220345251324494. 10.1177/00220345251324494 PMC12209541 40102739

[17] WHO and WHO , “Ending Childhood Dental Caries: Implementation Manual,” (2019), Implementation Manual [E‐book] 28 January 2020 [cited 2024 21 August], https://www.who.int/publications/i/item/ending‐childhood‐dental‐caries‐who‐implementation‐manual.

[18] A. T. M. Dao , L. G. do , N. Stormon , H. V. Nguyen , and D. H. Ha , “Key Mediators Reducing Socioeconomic Inequality in Early Childhood Caries,” JDR Clinical & Translational Research (2025): 23800844251365536, 10.1177/23800844251365536. PMC13232583 40931459

[19] A. T. M. Dao , L. G. Do , N. Stormon , H. V. Nguyen , and D. H. Ha , “G‐Computation Quantifying Caries Reduction by World Health Organization Sugar Limits in Children,” Journal of Dental Research (2026): 00220345251406559, 10.1177/00220345251406559. PMC13334843 41562134

[20] S. Gupta , “Intention‐To‐Treat Concept: A Review,” Perspectives in Clinical Research 2, no. 3 (2011): 109–112. 21897887 10.4103/2229-3485.83221 PMC3159210

[21] L. G. Do , J. A. Scott , W. M. Thomson , et al., “Common Risk Factor Approach to Address Socioeconomic Inequality in the Oral Health of Preschool Children–A Prospective Cohort Study,” BMC Public Health 14 (2014): 429. 24885129 10.1186/1471-2458-14-429 PMC4039048

[22] L. G. Do , D. H. Ha , L. K. Bell , et al., “Study of Mothers' and Infants' Life Events Affecting Oral Health (SMILE) Birth Cohort Study: Cohort Profile,” BMJ Open 10, no. 10 (2020): e041185. 10.1136/bmjopen-2020-041185 PMC7590353 33099500

[23] Australia NHMRC and NewZealand MOH , “Nutrient Reference Values for Australia and New Zealand, Including Recommended Dietary Intakes,” (2006), https://www.eatforhealth.gov.au/nutrient‐reference‐values/nutrients/dietary‐energy.

[24] M. Kuhn , Applied Predictive Modeling (Springer, 2013).

[25] A. T. M. Dao , L. G. Do , N. Stormon , H. V. Nguyen , and D. H. Ha , Key Mediators Reducing Socioeconomic Inequality in Early Childhood Caries (JDR Clinical & Translational Research, 2025). 10.1177/23800844251365536 PMC13232583 40931459

[26] M. Ferraro , A. R. Vieira , and F. Seymen , “Explaining Gender Differences in Caries: A Multifactorial Approach to a Multifactorial Disease,” International Journal of Dentistry 2010 (2010): 286–290. 10.1155/2010/649643 PMC2840374 20339488

[27] J. S. Ra and M. Park , “Sex‐Based Differences in Factors Associated With Sugar‐Sweetened Beverage Consumption Among Korean High School Students,” Frontiers in Nutrition (Lausanne) 9 (2022): 907922. 10.3389/fnut.2022.907922 PMC9237550 35774547

[28] Y.‐T. Lin , C.‐C. Chou , and Y.‐T. J. Lin , “Caries Experience Between Primary Teeth at 3–5 Years of Age and Future Caries in the Permanent First Molars,” Journal of Dental Sciences 16, no. 3 (2021): 899–904. 34141103 10.1016/j.jds.2020.11.014 PMC8189882

[29] L. Andrew , R. Wallace , N. Wickens , and J. Patel , “Early Childhood Caries, Primary Caregiver Oral Health Knowledge and Behaviours and Associated Sociological Factors in Australia: A Systematic Scoping Review,” BMC Oral Health 21, no. 1 (2021): 521. 34645446 10.1186/s12903-021-01887-4 PMC8513214

[30] P. D. P. Marsh , “Microbiology of Dental Plaque Biofilms and Their Role in Oral Health and Caries,” Dental Clinics of North America 54, no. 3 (2010): 441–454. 20630188 10.1016/j.cden.2010.03.002

[31] L. R. Costa , M. V. Vettore , L. N. Quadros , et al., “Socio‐Economic Status, Psychosocial Factors, Health Behaviours and Incidence of Dental Caries in 12‐Year‐Old Children Living in Deprived Communities in Manaus, Brazil,” Journal of Dentistry 133 (2023): 104504. 37019267 10.1016/j.jdent.2023.104504

[32] S. Antonoplis , “Studying Socioeconomic Status: Conceptual Problems and an Alternative Path Forward,” Perspectives on Psychological Science 18, no. 2 (2023): 275–292. 35981108 10.1177/17456916221093615 PMC10018062

[33] Y. Matsuyama , A. Isumi , S. Doi , and T. Fujiwara , “Persistent Poverty and Child Dental Caries: Time‐Varying Exposure Analysis,” Journal of Epidemiology and Community Health 77, no. 10 (2023): 670–675. 37468269 10.1136/jech-2022-220073

[34] R. G. Vieira‐Andrade , I. A. Pordeus , M. L. Ramos‐Jorge , et al., “Risk Indicators of Untreated Dental Caries Incidence Among Preschoolers: A Prospective Longitudinal Study,” Brazilian Oral Research 36 (2022): 12. 10.1590/1807-3107bor-2022.vol36.0064 36507751

[35] H. Al Tunaiji , J. C. Davis , M. A. Mansournia , and K. M. Khan , “Population Attributable Fraction of Leading Non‐Communicable Cardiovascular Diseases due to Leisure‐Time Physical Inactivity: A Systematic Review,” BMJ Open Sport & Exercise Medicine 5, no. 1 (2019): e000512. 10.1136/bmjsem-2019-000512 PMC6539142 31191969

[36] H. Al Tunaiji , J. C. Davis , D. C. Mackey , and K. M. Khan , “Population Attributable Fraction of Type 2 Diabetes due to Physical Inactivity in Adults: A Systematic Review,” BMC Public Health 14, no. 1 (2014): 469. 24885278 10.1186/1471-2458-14-469 PMC4083369

[37] J. Zhang , X. Guo , Z. Lu , et al., “Cardiovascular Diseases Deaths Attributable to High Sodium Intake in Shandong Province, China,” Journal of the American Heart Association 8, no. 1 (2019): e010737. 30563415 10.1161/JAHA.118.010737 PMC6405719

[38] Australian Bureau of Statistics, “Births, Australia, 2023 [Internet],” 2024, https://www.abs.gov.au/statistics/people/population/births‐australia/2023.

[39] AIHW , “Patient Demographics,” (2025), [cited Accessed 23 May 2025], https://www.aihw.gov.au/hospitals/topics/admitted‐patient‐care/patient‐demographics?utm_source=chatgpt.com.

[40] R. Pawson and N. Tilley , Realistic Evaluation (Sage, 1997).

[41] N. R. Cook , J. A. Cutler , E. Obarzanek , et al., “Long Term Effects of Dietary Sodium Reduction on Cardiovascular Disease Outcomes: Observational Follow‐Up of the Trials of Hypertension Prevention (TOHP),” BMJ 334, no. 7599 (2007): 885–888. 17449506 10.1136/bmj.39147.604896.55 PMC1857760

[42] F. P. Cappuccio , “Salt and Cardiovascular Disease,” BMJ 334, no. 7599 (2007): 859–860. 17463420 10.1136/bmj.39175.364954.BE PMC1857801

[43] R. Gilbert , G. Salanti , M. Harden , and S. See , “Infant Sleeping Position and the Sudden Infant Death Syndrome: Systematic Review of Observational Studies and Historical Review of Recommendations From 1940 to 2002,” International Journal of Epidemiology 34, no. 4 (2005): 874–887. 15843394 10.1093/ije/dyi088

