# Supplementary material for: Translating Observational Data Into Public Health Action: Reducing Early Childhood Caries Burden Related to Socioeconomic Disparities
Source: Community Dent Oral Epidemiol. 2026 Mar 23;54(4):471–9. doi: 10.1111/cdoe.70064 (PMC13428044; doi:10.1111/cdoe.70064)

Appendix:

Translating Observational Data into Public Health Action:

Reducing early childhood caries burdens related to socioeconomic disparities.

An T M Dao^1,2,3^, Loc G Do^1^, Helena Schuch^1^, Huy V Nguyen ^3,2,4^ Diep H Ha^1^

1. *School of Dentistry, Faculty of Health and Behavioural Sciences, The University of Queensland, Australia*
2. *Cuu Long University, Vietnam*
3. *Division of Epidemiology, Department of Population and Quantitative Health Sciences, UMass Chan Medical School*
4. *Health Innovation and Transformation Centre, Federation University Australia, Ballarat, Victoria, Australia*

**Corresponding author.**

An Thi Minh Dao

School of Dentistry, Faculty of Health and behavioural Sciences, The University of Queensland, Australia

288 Herston Road, Brisbane, Queensland, 4006, Australia

Tel: +61 0404313898

Email: [t.dao@uq.edu.au](mailto:t.dao@uq.edu.au)

ORCID ID

Thi Minh An DAO. [https://orcid.org/0000-0002-1942-3009](https://orcid.org/0000-0002-1942-3009?lang=en)

Tables. 02

Figure. 04

Table 1. Twenty longitudinal observational studies examining association between SES and dental caries (2012-2024)

| **Articles** | **Primary exposures** | **Third variables** | **Outcomes** | **Age of outcome measurements** |
| --- | --- | --- | --- | --- |
| **Australia** |  |  |  |  |
| [1] | Childhood caries interacts with family SES. | Childhood caries Tooth-brushing at age 30 and child sex. | DMFT | 13, 30 yrs |
| [2] | Family construct, socioeconomic status, family composition, family function, parental health status and health behaviours, social supports, physical safe, culture. | Gender, indigenous status, main language spoken at home, age, period. | dmft & DMFT | 2-16yrs |
| [3] | Sociodemographic (gender, education level, parity, number of children in the household, housing area per person, and living district)  Oral health related (parents’ knowledge of self and child’s oral health)  Diet (three-day food record)  Child’s OHI-S at baseline and follow-up | None | DMFT ↑ | 7-8, 9-11y |
| [4] | Maternal education and household income. | Children’s consumption of sugary food and sugary drinks | dmfs | 5-9yrs |
| **Brazil** |  |  |  |  |
| [5] | Family income trajectories at birth, 4, 11, 15, and 18 yrs | Mother’s education trajectories measured at 3 points (participants’ birth-year and ages 15 and 18); toothbrushing frequency trajectories at ages of 6, 12, 15, and 18 years; dental visiting at ages of 6, 12, and 15; dental caries in primary dentition at age of 6, and birth weight | DFMT | 6,12,18yrs |
| [6] | Baseline conducted in 2010 in children aged 1-5 yrs  Community factors: Present of cultural community centre  Sociodemographic factors: sex, skin colour, age, family income, maternal education, household crowding  Behavioural psychosocial and biological factors:  Tooth brushing, dental attendance,  parents’ perception of child oral health, dental plaque, and dental caries | None | DMFT | 8-12yrs |
| [7] | Mother’s oral health, trajectories of family income, and maternal education. | Mother’s oral health child’s sex, age, daily intake of sugar snacks, mother’s daily sugar intake, toothache history, dental visit, child’s oral health status at baseline. | ECC ↑ | 1-3,  4-<6yrs |
| [8] | Contextual level: neighbourhood’s mean income  Sociodemographic: sex, household income, maternal education  Oral clinical and subjective variables: use dental service, types of service, dental plaque, gingival bleeding, toothache, self-perception of oral health | None | DMFT | 12,14,17.5  yrs |
| [9] | Monthly household income | Child health problem, PUFA index, history of toothache, socioeconomic factors, baseline untreated dental caries, oral health quality of life (OHRQoL) | ECC↑ | 2-3yrs,  3-5yrs |
| [10] | SES, psychosocial factors, toothbrushing, sugar consumption and sedentary behaviour. | None | DMFS↑ | 12-13 yrs |
| [11] | Maternal education, family income, parental perception on child oral health, severe dental caries experience | None | DMFS ↑ | 1-<5yrs +7 |
| [12] | Socioeconomic disparities: child's sex, maternal skin colour/ race, maternal education, family monthly income measure for 48-month follow-up | None | dmft | 4-6yrs |
| [13] | Child’s sex, age, skin colour, parent’s education, employment status, family structure, household income, children’s behavioural and psychosocial characteristics, frequency of toothbrushing with fluoridated toothpaste, consumption of refined sugars or other simple carbohydrates, parents’ perception of children’s oral health, children’s guardians’ social capital. | None | DMFS ↑, dmfs↑ | 1-5 yrs,  + 2y & 10 |
| [14] | Gender, child's skin colour, school type, father's and mother's occupation, mother's and father's education, beneficiary of state family allowance. | None | DMFS ↑, dmfs↑ | 11yrs+4 |
| **US** |  |  |  |  |
| [15] | Added SSB intake, pure juice beverage intake at 36 months.  Mother’s age, DMFS, brushing frequency, smoking habits, education level, annual family income, and household size at baseline. | None | ECC | 3yrs |
| [16] | Mutans streptococci (MS), lactobacilli, candida species, salivary cortisol, and salivary IgA, child’s eating and drinking habits, snacking choices, sippy cup use, and the type and number of beverages and snacks consumed by the children; breastfeeding and formula-feeding practices and the children’s oral hygiene regimens, child and family stress, children’s and parents/primary caregivers’ age, race, ethnicity, and gender; parent/primary caregiver education, occupation, and income. | None | New ECC | 3-5yrs |
| [17] | SES, daily fluoride intake, frequency of milk intake, amount of SSB intake, and lower age 17 dental caries counts. |  | DMFS↑ | 17-23yrs |
| **Japan** |  |  |  |  |
| [18] | SES (parents’ educational attainment) | Child sex and age | ECC ↑ | 2.5,3.5,4.5,5.5yrs |
| [19] | Annual household income, material deprivation or payment difficulties for lifeline utilities | Child’s age, child’s sex, birth order, mother and father’s age, mother, and father’s educational attainment.  Time-varying confounders: caregivers’ psychological distress, living with grandparents, and marital status of parents. | dmft↑ & DMFT↑ | 6,7,9,11 yrs |
| **Portugal** |  |  |  |  |
| [20] | SES: area of residence; parental place of birth, educational level, and occupation; number of people in household; siblings’ existence; child’s birth order and place of birth  Oral hygiene and dental visit: tooth-brushing; age of tooth-brushing; help during toothbrushing; dentifrice type; age of first dental visit; frequency of dental visits,  Diet: breastfeeding habits after the first year of life; number of current daily meals and how often sweets are consumed | None | ECC | 5 and 5.5yrs |
| dmft, decayed, missing, or filling teeth (primary dentition); DMFT, decayed, missing, or filling teeth (permanent dentition); ECC, early childhood caries; m, month; y, year; yrs: years old; SES, socioeconomic status; IQ; SSBs, sugar-sweetened beverages; Traj, trajectories; FSI: Free Sugar Intake; RR: Retention Rate; SNP: Single Nucleotide Polymorphisms; OHRQoL: Oral Health Quality of Life; PUFA: Visible pulp exposure, Ulceration of the oral mucosa due to root fragments, Fistula, and [Abscess](https://www.iosrjournals.org/iosr-jdms/papers/Vol17-issue4/Version-18/C1704180914.pdf) | | | | |

Table 2. Alignment types of the Directed Acyclic Graphs (DAGs) and statistical methods

|  | Completely not use time varying | | | | | Fully use-time varying | | | Cross-sectional data | |  |
| --- | --- | --- | --- | --- | --- | --- | --- | --- | --- | --- | --- |
| Statistics | Cau | Med | TVC | TV | Sub_Total | TCF | TVC | Sub_Total | Cau | Sub_Total | Total |
|  |  |  |  |  |  |  |  |  |  |  |  |
| Only descriptive statistics | 1 | 0 | 0 | 0 | 1 | - | - | - | - | - | 1 |
| Counterfactual approach (TMLE) | 0 | 0 | 1 | 0 | 1 | - | - | - | - | - | 1 |
| GLM | 3 | 0 | 0 | 0 | 3 | - | - | - | 2 | 2 | 5 |
| GLM*I | 0 | 0 | 0 | 1 | 1 | - | - | - | 1 | 1 | 2 |
| MER | 0 | 0 | 0 | 1 | 1 | 0 | 1 | 1 | 1 | 1 | 3 |
| ML+GLM | 1 | 0 | 0 | 0 | 1 | - | - | - | - | - | 1 |
| PR | 2 | 0 | 0 | 0 | 2 | - | - | - | 1 | 1 | 3 |
| SEM | 0 | 1 | 0 | 0 | 1 | - | - | - | - | - | 1 |
| GEE | - | - | - | - | - | 2 | 0 | 2 | - | - | 2 |
| SVA | - | - | - | - | - | - | - | - | 1 | 1 | 1 |
| Total | 7 | 1 | 1 | 2 | 11 | 2 | 1 | 3 | 6 | 6 | 20 |

Light grey: applying statistical causal inference approach

Dark grey: pathway analysis without statistical causal inference approach

Na: not applied modelling

ML: Multi-Level

GLM: Generalise Linear Regression

GLM*I: Generalise Linear Regression with interaction

PR: Poisson Regression

GEE: Generalised Equation Estimation

MER: Mixed-Effect Regression

SEM: Structural Equation Modelling

SVA: Survival Analysis

TMLE: Targeted Maximum Likelihood Estimator

(-) Not applicable

Figure 1. Directed Acyclic Graphic, visualising relationship between Free Sugar Intake age two and Early Childhood Caries at five


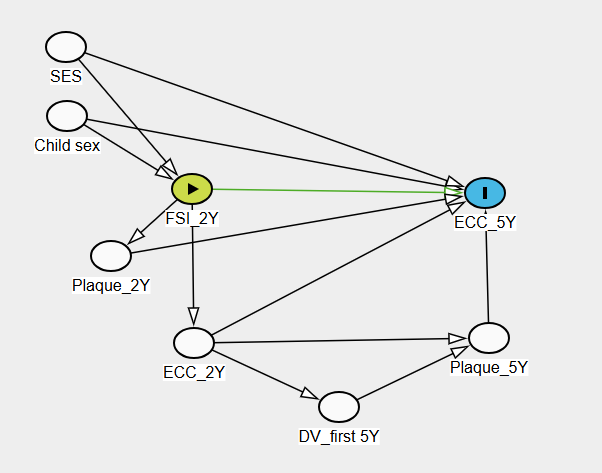


SES: Socioeconomic status

FSI_#y: Free sugar in take at year #

Plaque_#y: Plaque index at age #

ECC_#y: Early childhood caries at age #

DV_first 5y: Number of dental visits during the first five years of life

Figure 2. G-computation framework,

estimating Absolute Reduction and Attributable Fraction among Exposure


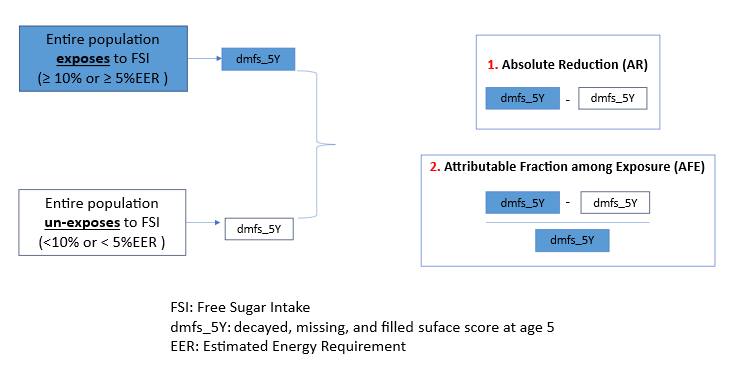


Figure 3. SES relevant items used in longitudinal observational studies predicting dental caries

from 2012-2024

Figure 4. Missing, filled surfaces (dmfs) scores reduction at age five in the conditions of

of Free Sugar Intake age two below 10% and 5% of total estimated energy requirements.


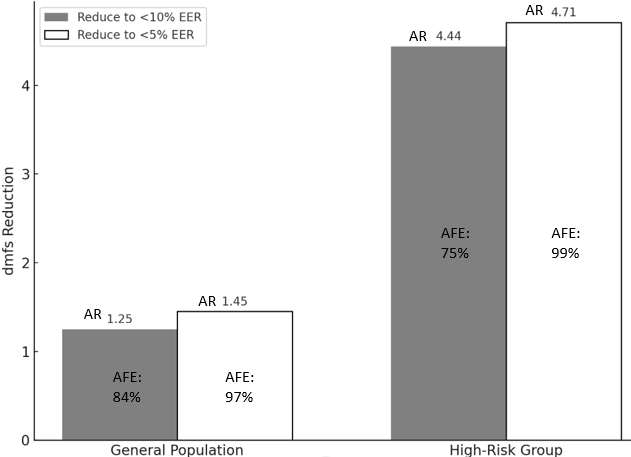

Supplement: Supplementary file 1 — Table S1: Twenty longitudinal observational studies examining association between SES and dental caries (2012–2024). TABLE S2: Alignment types of the Directed Acyclic Graphs (DAGs) and statistical methods. FIGURE S1: Directed Acyclic Graphic, visualising relationship between Free Sugar Intake age two and Early Childhood Caries at five. FIGURE S2: G‐computation framework, estimating Absolute Reduction and Attributable Fraction among Exposure. FIGURE S3: SES relevant items used in longitudinal observational studies predicting dental caries from 2012 to 2024. FIGURE S4: Missing, filled surfaces (dmfs) scores reduction at age five in the conditions of Free Sugar Intake age two below 10% and 5% of total estimated energy requirements. [file CDOE-54-471-s001.docx]
